# Research training needs on HIV and oral health among dentists in resource limited settings: A cross-sectional study

**DOI:** 10.21203/rs.3.rs-5923967/v1

**Published:** 2025-03-31

**Authors:** William Buwembo, Laban Muteebwa, Adriane Kamulegeya, Ian G. Munabi, Arabat Kasangaki, Aloysius G. Mubuuke, Katumba Sentongo, Catherine Lutalo Mwesigwa, Annet Kutesa, Lauren L. Patton, Ronald P. Strauss, Fred Collins Semitala

## Abstract

**Background:** Oral health among people living with Human Immunodeficiency Virus (HIV) in Uganda is under-researched, despite its critical impact on their overall health and quality of life. This gap is partly attributed to limited capacity in human resources and research infrastructure. This study aimed to evaluate the HIV/oral health research training needs among dentists in Uganda. **Methods:** We conducted a cross-sectional study among 86 dentists licensed to practice in Uganda. A self-administered online questionnaire, adapted from the Hennessey-Hicks training needs analysis tool, was used to collect data. The questionnaire included four rating scales to assess HIV/oral health research training needs by comparing the perceived importance of specific competencies to the current performance ratings provided by participants. Performance improvement was analyzed using two rating scales to determine whether the identified training needs could be better addressed through organizational changes or individual training. Data analysis was performed using STATA version 17.0. **Results:** Between September and October 2024, 86 dentists participated in the study. The median age of participants was 32 years (interquartile range (IQR): 27–38), with 59.3% being male. Significant HIV/oral health research training needs were identified, with a median difference score between perceived importance and current performance ratings of 1.3 (IQR: 0.5–2.2) and a P-value of <0.001. Both organizational changes and individual training approaches were similarly rated as important in addressing these training needs, with median scores of 5.9 (IQR: 5.1–6.6) and 6.1 (IQR: 5.2–6.7), respectively (P-value = 0.108). **Conclusion:** The findings highlight significant HIV/oral health research training needs among dentists in Uganda. Addressing these needs requires a combined approach that integrates individual training with organizational support initiatives.

## Background

Globally there are over 39.9 million people living with Human Immunodeficiency Virus (HIV), with more than 65% live in Sub-Saharan African[1]. In Uganda, approximately 1.5 million people living with HIV (PLHIV) of whom 94% are on lifelong antiretroviral therapy (ART) and 94% are virally suppressed[2]. The introduction of ART has significantly improved the life expectancy of PLHIV, bringing it closer to that of the general population[3]. Despite these advancements, PLHIV face a disproportionate burden of oral health conditions, which negatively impact their quality of life [4]. The oral cavity remains a common site for opportunistic infections in PLHIV, with conditions such as periodontal diseases, oropharyngeal candidiasis, oral warts, oral hairy leukoplakia, and Kaposi sarcoma frequently reported, even among those on ART [5]. Historically, oral conditions were used for HIV diagnosis, clinical staging, and identifying AIDS in resource-limited settings[4]. Emerging evidence highlights significant differences in the oral microbiome of PLHIV compared to HIV-negative individuals [6]. A recent large-scale survey in Uganda found that over 66% of 4,449 PLHIV had periodontal disease, with nearly half (48.2%) experiencing moderate to severe clinical attachment loss[7] which is further exacerbated by aging and prolonged ART use[7]. These findings underscore the critical need to address oral health co-morbidities in an aging population of PLHIV on long term treatment.

Moreover, oral health research among PLHIV remains neglected in Uganda, where oral health is not integrated into the national HIV programing or policies [8]. This gap persists despite the routine clinic visits PLHIV make for ART refills and evaluations, presenting opportunities to address their oral health concerns. The lack of focus on oral health research denies PLHIV access to evidence-based interventions that could enhance their quality of life. Whereas Ugandan scientists have made significant contributions to HIV-related research[9, 10], including through international collaborations and capacity-building initiatives, HIV/oral health research remains under-represented[11]. Barriers such as weak institutional support, inadequate funding, focus on service delivery, and lack of protected time for research limit the involvement of oral health specialists like dentists in such studies[12, 13]. Furthermore, the absence of clear policy guiding HIV/oral health care excludes dentists from routine oral health care for PLHIV[8]. To address these gaps, we aimed to identify the HIV/oral health research training needs among dentists in Uganda. By pinpointing specific skills gaps, this study seeks to inform the development of tailored training programs that could empower dentists and other oral health practitioners to contribute to HIV/oral health research, ultimately improving care for PLHIV.

## Methods

#### Study design and setting

This study employed a cross-sectional design to assess HIV/oral health research training needs among dentists in Uganda. Uganda has an estimated population of 45 million, projected to reach 85 million by 2040[5]. With a gross domestic product (GDP) of approximately $40 billion in 2023 and a GDP per capita estimated at $850 USD, Uganda is classified as a low-income country. The country is in the generalized HIV epidemic phase, with a prevalence of 5.1% (ranging from 0.5% to 14.1% across districts) (16). Approximately 94% of PLHIV in Uganda are on lifelong ART, which is provided free of charge to the PLHIV[2].

Uganda has four recognized dental schools that train graduates in dentistry, awarding the Bachelor of Dental Surgery (BDS) degree. Currently, approximately 300 dentists holding at least a BDS degree are licensed to practice in Uganda by the Uganda Medical and Dental Practitioners Council (UMDPC), the statutory licensing body for medical and dental professionals in the country. This setting provided a unique opportunity for us to evaluate the training needs of dentists in HIV/oral health research within a low-resource context where oral health is not yet integrated into HIV care and policy frameworks.

#### Study population, inclusion and exclusion criteria

We enrolled 86 dentists licensed to practice in Uganda. One was eligible if s/he:1. holds a Bachelor of Dental Surgery (BDS) or equivalent degree with a valid license to practice in Uganda, as certified by the UMDPC; 2. actively practicing dentistry in Uganda during the data collection period. Dentists were excluded if they were unreachable through the contact information provided in the UMDPC registry or had relocated from Uganda before the study commenced.

#### Sample size estimation and sampling procedure

We enrolled 86 dentists through consecutive sampling by the available registry of licensed dentists maintained by UMDPC until the required sample size was reached. Based on the target population, we calculated the sample size using a sample size formula for mean in one group previously reported by Beth and Robert 2004[14]. The formula N= was used, where $Z_{\alpha/2}$ (1.96) is the Z value corresponding to 5% level of significance, SD is the standard deviation of the differences obtained from a pilot (1.23). To ensure 90% power for detecting an effect size ($\mu_1$-$\mu_0$) of at least 0.5, and accounting for 25% non-response, the total estimated sample size was 86.

#### Participant recruitment and data collection procedure

We contacted eligible participants via email, phone calls and, and in-person visits. The initial contact introduced the study's objectives and procedures, with an invitation to participate voluntarily.  For those unreachable though initial contact methods, a follow-up was conducted using alternative channels or known professional networks.  unresponsive or unavailable dentists after three attempts were excluded.

#### Data collection tools

We used a structured questionnaire to collect data on demographic characteristics including age, sex, highest academic qualification and institutional affiliation, prior research training and experience, and responses to a comprehensive training needs assessment. The HIV/oral health research training needs were evaluated using the World Health Organization (WHO)-adopted Hennessy-Hicks Training Needs Analysis Questionnaire[15]. This validated tool consists of 30 questions organized into five key domains of research activities: 1.   Communication and teamwork (6 questions); 2. Clinical tasks (6 questions); 3. Administrative tasks (3 questions); 4. Supervisory tasks (6 questions); and 5. General research activities (9 questions).  These domains assess the competencies required for an effective HIV/oral health researcher. To tailor the questionnaire for this study, an additional domain specific to HIV/ oral health tasks was included, comprising  three questions[16]. This domain evaluated competencies specific to oral health professionals managing PLHIV. Evidence supports the robustness of the Hennessy-Hicks tool, even with the inclusion of up to 10 additional items, without compromising its validity[15].

The modified questionnaire used a four-scale rating system to assess various dimensions of training needs, with ratings ranging from 1 to 7, where higher scores represented greater importance or higher levels of performance. The four scales included: 1. Importance rating – defined as the participant's perceived importance of a specific task for becoming a competent HIV/oral health researcher; 2. Current performance rating – defined as the participant's self-assessed level of performance for the task at their current workstation; 3.Organizational change rating – defined as the extent to which addressing the training need would require organizational-level adjustments or interventions at the participant's workstation; and 4. Individual training rating – defined as the extent to which addressing the training need would require individual-level capacity building through training and mentorship.

#### Study outcomes

The HIV/oral health research training need was the primary outcome and was assessed as the difference between the average importance rating and the average current performance rating, and a score above zero was considered as having a training need [15].  The scope of performance improvement was assessed using two rating scales where participants were asked to rate whether the training need can better be addressed by organizational changes or individual training[15]. The approach with a higher average score was regarded as the most appropriate approach to address the training needs

#### Data analysis

We analyzed the data using STATA version 17.0 (StataCorp LLC, Texas, USA). Continuous variables were summarized with the median and interquartile range (IQR), while categorical variables were summarized as frequencies and percentages. The difference score between the importance and current performance ratings was summarized with the median and IQR and displayed in a box plot. The significance of the difference score from zero was assessed using a one-sample sign test, and a one-sided P-value was reported.

Organizational change and individual training rating scores were summarized with the median and IQR and presented in box plots. Differences in paired medians were assessed using the Mann-Whitney U-test, and a two-sided P-value was reported. Additionally, differences in the medians for each domain (general research activities, communication/teamwork, clinical tasks, administration, supervisory tasks, and HIV/oral health-specific tasks) were assessed using the Mann-Whitney U-test, and a two-sided P-value was reported.

### Results

#### Demographic characteristics of study participants

Between September and October 2024, 86 dentists participated in the study. The median age of participants was 32 (IQR: 27-38) years. Slightly more than half were males (59.3%) and worked in private sector (58.1%). The majority (81.4%) held a Bachelor's degree as their highest level of education, with approximately two-thirds (60.5%) having attained the degree within the past five years (Table 1).

**Table 1: Characteristics of Dentists that participated in the needs assessment survey**

| Variable | Categories | Frequency (N=86) | Percentage (%) |
|---|---|---|---|
| Age (years) | Median (IQR) | 32 (27, 38) | |
| Sex | Male | 51 | 59.3 |
| | Female | 35 | 40.7 |
| Institution of affiliation | Public | 33 | 38.4 |
| | Private | 50 | 58.1 |
| | NGO* | 3 | 3.5 |
| Current position/ appointment | Faculty | 4 | 4.7 |
| | Dentists | 68 | 79.1 |
| | Post-Graduate student | 6 | 7.0 |
| | Research/Program officer | 4 | 4.6 |
| | Others | 4 | 4.6 |
| Highest academic qualification | Bachelor's degree (BDS) | 70 | 81.4 |
| | Master's degree | 16 | 18.6 |
| Period since graduation | ≤5 years | 52 | 60.5 |
| | 6-10 years | 23 | 26.7 |
| | >10 years | 11 | 12.8 |

**NGO-Non-Governmental Organization*

#### Previous research-related experience

Slightly more than half of the study participants (57.0%) had received training in Epidemiology, and a similar proportion (55.8%) had undergone training in Biostatistics. Two-thirds of the participants reported having participated in health-related research activities, with 42.1% currently engaged in health-related research activities. Only 15.1% reported to have ever published in a peer-reviewed journal, with a median number of published papers of 2 (IQR:1-3) and a range of 1-10 publications. Furthermore, only 3 of the 13 participants who had published papers reported being the first author of any of their publications (Table 2).

**Table 2: Research-related training and experiences of dentists who participated in the needs assessment survey**

| Variable | Categories | Frequency (N=86) | Percentage (%) |
|---|---|---|---|
| Ever had training in Epidemiology | Yes | 49 | 57.0 |
| | No | 37 | 43.0 |
| Epidemiology training mode* | Course unit at bachelor's degree | 18 | 36.7 |
| | Course unit at master's degree | 14 | 36.7 |
| | Course unit at PhD | 1 | 2.0 |
| | Short course | 14 | 28.6 |
| | Specialized master's degree | 2 | 4.0 |
| Ever had training in Biostatistics | Yes | 48 | 55.8 |
| | No | 38 | 44.2 |
| Biostatistics training mode** | Course unit at bachelor's degree | 16 | 33.3 |
| | Course unit at master's degree | 16 | 33.3 |
| | Course unit at PhD | 1 | 2.1 |
| | Short course | 14 | 29.2 |
| | Specialized master's degree | 1 | 2.1 |
| Participation as a core team of a health-related research | Yes | 57 | 66.3 |
| | No | 29 | 33.7 |
| Number of research projects worked on | Median (IQR) | 2 (1, 4) | |
| Number of research projects initiated by participant | Median (IQR) | 0 (0, 1) | |
| Current participation in ongoing research*** | Yes | 24 | 42.1 |
| | No | 33 | 57.9 |
| Current position on a research project# | Investigator | 9 | 37.5 |
| | Dentist | 6 | 25.0 |

| | | | |
|---|---|---|---|
| | Research assistant | 5 | 20.8 |
| | Others | 4 | 16.7 |
| Ever published in a peer-reviewed journal | Yes | 13 | 15.1 |
| | No | 73 | 84.9 |
| Number of published scientific papers | Median (IQR) | 2 (1, 3) | |
| Publication as first author## | Yes | 3 | 23.1 |
| | No | 10 | 76.9 |
| Ever participated in grant writing | Yes | 11 | 12.8 |
| | No | 75 | 87.2 |
| Number of grant applications written | Median (IQR) | 1 (1, 2) | |
| Previous training on oral health in PLHIV$ | Yes | 57 | 66.3 |
| | No | 29 | 33.7 |
| Interest in HIV/Oral health research | Median (IQR) | 8 (7, 10) | |

**N=49, **N=48, ***N=57, #N=24, ##N=13, $PLHIV-People Living with HIV*

**Training needs assessment**

The overall median of training needs score was 1.3 (IQR: 0.5- 2.2), which was statistically greater than zero (P value <0.001). The Median (IQR) training needs scores for specific domains were as follows: Research activities: 1.2 (IQR: 0.4–2.3), Communication/Teamwork: 1.0 (IQR: 0.2–1.8), Clinical Tasks: 1.2 (IQR: 0.4–2.2). Each of these scores was statistically significant (P < 0.001). Similarly, the median training needs scores for other areas were: Administration: 1.3 (IQR: 0.3–2.0), Management/Supervisory Tasks: 1.3 (IQR: 0.5–2.3), HIV/Oral Health-Specific Tasks: 1.5 (IQR: 0.7–2.3). All these scores were also statistically significant (P < 0.001) (Figure 1).

**Scope of Performance Improvement on Overall HIV/Oral Health Training Needs**

Study participants indicated that addressing HIV/oral health research training needs requires both individual training and organizational change. The median (IQR) score for individual training was 6.1 (5.2–6.7), while the median (IQR) score for organizational change was 5.9 (5.1–6.6) (Figure 2). There was no statistically significant difference between the two paired medians (P = 0.108).

**Scope of performance improvement on the overall HIV/oral health training needs by competencies of an HIV/oral health researcher**

The participants expressed preference for addressing the training needs related to research activities, communication/teamwork and management/supervisory tasks by individual training as compared to organizational change with P values of 0.015, 0.025 and <0.001 respectively. For training needs related to clinical, administrative and HIV/oral health-specific tasks, participants rated both individual training and organizational change approaches similarly (Table 3).

**Table 3: Comparison of median of organizational change and individual training rating score of the key competencies of a HIV/oral health researcher**

| Tasks | Organizational change<br>Median (IQR) | Individual training<br>Median (IQR) | Mann-Whitney U-Test P value |
|---|---|---|---|
| Research activities | 6.0 (5.0, 6.7) | 6.3 (5.3, 6.8) | **0.015** |
| Communication/Teamwork tasks | 6.0 (5.2, 6.7) | 6.2 (5.0, 6.7) | **0.025** |
| Clinical tasks | 6.0 (5.2, 6.7) | 6.0 (5.2, 6.8) | 0.690 |
| Administration tasks | 6.0 (5.3, 6.7) | 6.3 (5.0, 6.7) | 0.351 |
| Management/Supervisory Tasks | 5.8 (4.8, 6.5) | 6.0 (5.0, 6.8) | **<0.001** |
| HIV/Oral health specific tasks | 6.3 (5.3, 7.0) | 6.3 (5.3, 7.0) | 0.771 |

### Discussion

## HIV/oral health research training needs

This study revealed significant HIV/oral health research training needs among dentists in Uganda, spanning core competences essential for oral health researchers working with PLHIV. Additionally, the dentists demonstrated a high interest in oral health research involving PLHIV, underscoring the necessity of addressing these needs to support research and improve care in this underserved area.

PLHIV in Uganda and other Sub-Saharan African countries face disproportionate burden of oral health complications despite wide spread ART coverage [7][17]. Addressing gaps in HIV/oral health research is critical to reducing health inequalities and improving quality of life for PLHIV. In Uganda, oral health practitioners and HIV care providers often work in isolation, and dental practice predominantly focuses on service delivery rather than research. [12]. University-based dentists/dental practitioners frequently face competing responsibilities, such as teaching and administration, which limit their research engagement. Challenges such as weak institutional support, inadequate funding, lack of research equipment, and limited mentorship further compound these barriers[12, 13]. To bridge these gaps, there is a need for a dedicated training program to build research capacity among dentists. Leveraging their demonstrated interest in HIV/oral health research, such programs should focus on equipping dentists/dental practitioners with necessary skills and providing mentorship opportunities. This approach will foster evidence-based practices and inform policy decisions, ultimately improving outcomes for PLHIV.

## Scope of performance improvement

The study highlighted the importance of both individual and organizational change in addressing HIV/oral health research training needs. Dentists rated both approaches highly, with no statistically significant difference between their median scores. This finding emphasizes the duo responsibility of individual competency development and institutional support in fostering an HIV/ oral research agenda

In Uganda, most dentists work in private clinical settings that often lack infrastructure to support research. These settings often have limited exposure to PLHIV, reducing practical experience in addressing their oral health needs. Additionally, oral health services are not integrated into larger HIV programming, missing opportunities for research and care [8]. On the other hand, dentists in academic institutions are often pre-occupied with teaching, and clinical and administrative duties, limiting their ability to engage in research [12, 18]. Organizational change, such as implementing research-supportive policies, improving access to resources, and dedicating time for research, is essential to overcoming these structural barriers.

Our findings also revealed low levels of formal research training among dentists. While two-thirds had prior research involvement, only half had received basic training in epidemiology and biostatistics, primarily through short courses. These competencies are crucial for conducting and publishing high-quality research. Notably, a dismal 15.1% of participants had published in peer-reviewed journals, and 10% had experience with grant writing. These gaps highlight the need for targeted individual training to enhance research skills and practical experience, enabling dentists to better address the unique oral health challenges faced by PLHIV. While postgraduate training includes research components, evidence suggests these are insufficient for sustaining research careers beyond academic theses [19]. Enhanced training opportunities, such as fellowships or long-term mentored programs, could significantly increase research output and improve scientific communication [20].

## Study limitation

This study relied on self-reported data collected through an online questionnaire, making it susceptible to recall and social desirability biases. To mitigate recall bias, participants were asked to reflect on the past 10 years, and the anonymous nature of the questionnaire likely reduced social desirability bias. While the HIV/oral health research needs assessment tool used in this study has been validated among Ugandan health workers, it was not specifically validated for dentists[21]. Nonetheless, the tool demonstrated high reliability (Cronbach’s alpha = 0.97) in this study population. The exclusive focus on graduate-level dentists may limit the generalizability of findings to lower-cadre dental practitioners

(below Bachelor's degree). Despite these limitations, the findings provide valuable insights into the training needs of dentists in Uganda.

### Conclusions

This study identified significant HIV/oral health research training needs among dentists in Uganda. Addressing these needs requires a dual approach involving both individual training and organizational support.

## Recommendations

To enhance HIV/oral health research and care in Uganda, academic institutions should integrate the unique oral health needs of PLHIV into curricula, establish structured research and mentorship programs, and provide in-service training opportunities. Health and academic institutions should develop research-focused units to foster interdisciplinary collaboration and offer dentists access to research resources. The Ministry of Health should standardize care through national guidelines, allocate resources for HIV/oral health care and research, and support continuous professional development for dentists/dental practitioners.

### Abbreviations

AIDS: Acquired Immunodeficiency Syndrome

ART: Antiretroviral Therapy

BDS: Bachelor of Dental Surgery

GDP: Gross Development Product

HIV: Human Immunodeficiency Virus

IQR: Interquartile Range

Mak-SOMREC: Makerere University School of Medicine Research Ethics Committee

NGO: Non-Governmental Organization

PLHIV: People Living with Human Immunodeficiency Virus

SD: Standard Deviation

UMDPC: Uganda Medical and Dental Practitioners Council

UNCST: Uganda National Council of Science and Technology

WHO: World Health Organization

## Declarations

### Ethical considerations

The study received ethical approval from the Makerere University School of Medicine Research Ethics Committee (Mak-SOMREC) under the reference number Mak-SOMREC-2024-980 and clearance from the Uganda National Council of Science and Technology (UNCST) under the reference number HS4603ES. All study participants provided informed consent prior to initiation of study procedures in compliance with UNCST guidelines.

### Acknowledgements

We acknowledge the study participants who invested time to participate in the study. We also acknowledge the contribution of Uganda Dental Association who supported participant mobilization activities.

### Funding

This study was funded by the Fogarty International Center (FIC) of the United States National Institutes of Health under award number D71TW012761 received by WB and FCS. We also appreciate the support from FIC and the National Institute on Mental Health (grant number D43TW010037) that supported LM as a trainee. The content is solely the responsibility of the authors and does not necessarily represent the official views of the National Institutes of Health. The funders had no role in the study design, data collection and analysis, decision to publish or preparation of the Manuscript

### Clinical trial number

Not applicable

### Consent for publication

Not Applicable

### Competing interests

The authors declare no competing interests

### Authors' contributions

FCS, WB, IGM and Adriane Kamulegeya (AK1) conceived the initial research idea. FCS, LM, WB, IGM, AK1 and Arabat Kasangaki (AK2) refined the research idea, drafted the initial proposal, and were involved in the whole research process through to the drafting of the manuscript.  LM, Annet Kutesa (AK3), AGM, KS, and CLM were instrumental in data collection and analysing the data. AK1, LLP and RPS critically read the initial draft of the manuscript. WB, LM and AK1 contributed equally as first authors. All co-authors reviewed and approved the final manuscript prior to submission.

#### Availability of dataset and materials

The datasets used during the analysis for this training needs assessment are available from the corresponding author upon reasonable request.

## Figures

P value for Hypothesis: Ho: Median score=0

Ha: Median score>0

**Figure 1**

A box plot of median and IQR for the Training needs scores

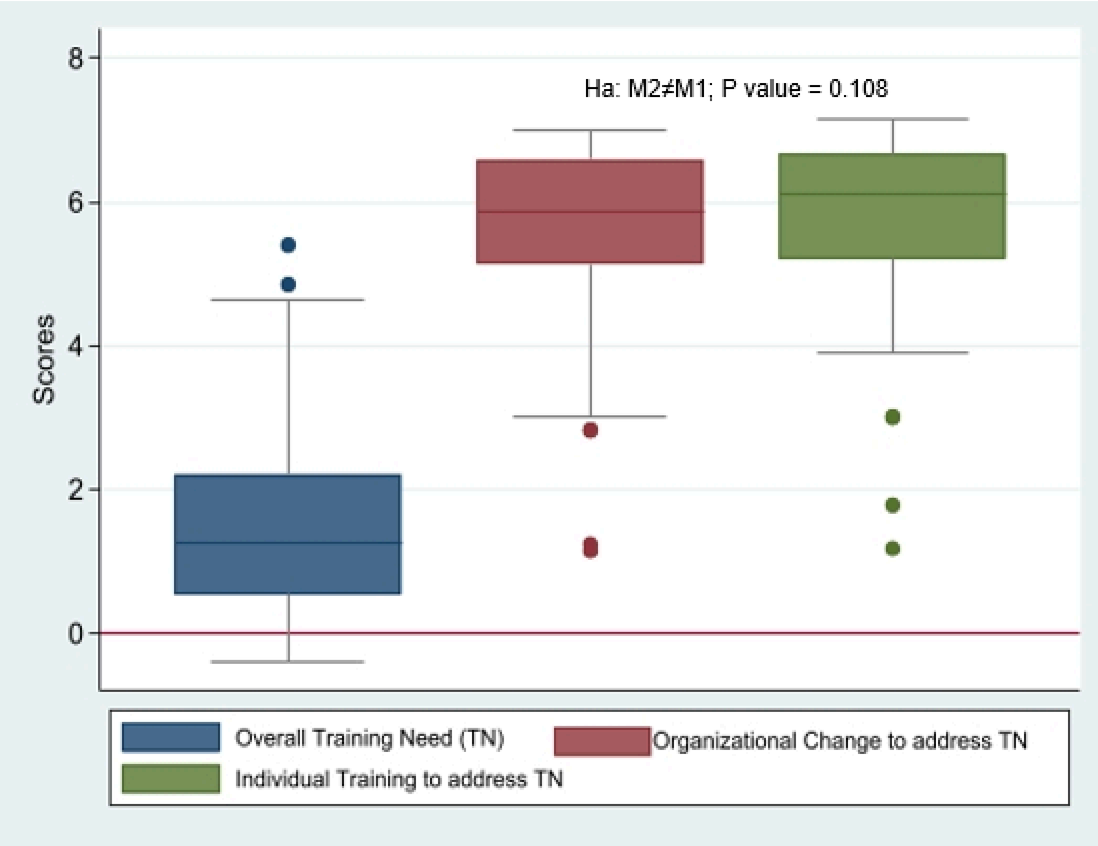

M1- Median of organizational change scores
M2- Median of individual training scores
Hypothesis: Ho: M1=M2
Ha: M2≠M1

### Figure 2

Participants’ rating of how to address the overall training needs (organizational change versus individual training)
